# Standards of referrals and admissions of critically ill patients to the ICU

**DOI:** 10.1186/cc12430

**Published:** 2013-03-19

**Authors:** A Ng

**Affiliations:** 1 Glasgow Victoria Infirmary Hospital, Glasgow, UK

## Introduction

The management of emergency medical admissions has been a subject of recent clinical incidents. There was a high percentage of patients that were referred to the ICU by staff in training, and 21% of referrals were made by junior doctors. Consultant physicians had no knowledge of the case in 57% of referrals.

## Methods

A prospective study of 21 cases of referrals and admissions to the ICU was conducted at the Glasgow Victoria Infirmary Hospital from 8 to 21 September 2012. A questionnaire was produced relating to the referrals, admissions, seniority involvement, cause of referral, and time of patient review by the ICU consultant after ICU admission. They were distributed to specialist registrars and the ICU consultants. All data were electronically recorded into an Excel database. Questionnaires that were not completely filled were further investigated using patient clinical notes and contact with medical staff. Information that may identify a patient or clinician was removed from the questionnaire for confidentiality purposes.

## Results

Twenty-one complete questionnaires were collected. Fifty-seven percent (12/21) of cases involved admission to the ICU. Nine percent of the cases involved contacting either a specialist registrar or ICU consultant intensivist for assistance in practical procedures. Of the patients admitted to the ICU, 33% (4/12) were from medical wards, 33% were admitted from A&E. Consultants were the most common professionals who referred patients to critical care (48%; 10/21). Fourteen percent of cases (3/21) involved the referral of patients into ICU by a junior doctor, but only one of the referrals was accepted by the ICU intensivist. Consultants referred or were aware of the referral in 71% (15/21) of cases. Of admissions, 58% (7/12) were accepted by the ICU consultant and the remaining by the specialist registrars. All accepted were acknowledged by the ICU consultant. After admission all of the patients were reviewed by the ICU consultant and the time of review after admission was on average 1 hour 23 minutes (25 minutes to 3 hours 45 minutes).

## Conclusion

There is still an issue with junior doctors referring patients to the ICU without the acknowledgement of consultant physicians, resulting in unnecessary admissions and decreased time that ICU trainees spend in the ICU. There are more appropriate ICU admissions when there is involvement with seniority. Contact with ICU staff to perform practical procedures outside the ICU and not about admissions should be explored further.
